# Un danger de la chirurgie thyroïdienne: le nerf laryngé inférieur non récurrent (cas clinique)

**DOI:** 10.11604/pamj.2020.35.9.20843

**Published:** 2020-01-10

**Authors:** Moustapha Ndiaye, Mame Sanou Diouf, Abdallah Witti, Mame Coumba Sarr, Ciré Ndiaye, Abdou Sy, Malick Ndiaye, Abdourahmane Tall

**Affiliations:** 1 Centre Hospitalier National Universitaire de Fann, Dakar, Sénégal; 2 Hôpital Général de Grand Yoff, Sénégal; 3 Hôpital Matlaboul Fawzeini de Touba, Touba, Sénégal; 4 Hôpital d’enfants de Diamniadio, Diamniadio, Sénégal

**Keywords:** Nerf laryngé récurrent, non récurrent, chirurgie thyroïdienne, Recurrent laryngeal nerve, non-recurrent, thyroid surgery

## Abstract

Les lésions du nerf laryngé inférieur représentent les complications les plus dévastatrices de la chirurgie thyroïdienne. L'identification du nerf laryngé inférieur est d'autant plus difficile que son trajet est atypique. Nous rapportons un cas de nerf laryngé inférieur droit non récurrent découvert à l'occasion d'une thyroïdectomie.

## Abstract (en)

Lesions of the inferior laryngeal nerve are the most devastating complications of thyroid surgery. Identifying the inferior laryngeal nerve is all the more difficult when its path is atypical. We report a case of right non-recurrent inferior laryngeal nerve detected during thyroidectomy.

## Introduction

Les lésions du nerf laryngé inférieur représentent les complications les plus dévastatrices de la chirurgie thyroïdienne [1]. Les variations anatomiques du nerf laryngé inférieur rendent difficile son identification et constituent un véritable danger chirurgical. Parmi ces variations anatomiques, nous avons le nerf laryngé inférieur non récurrent qui se détache du nerf vague et se dirige directement vers le larynx sans pour autant emprunter un trajet récurrent. Nous rapportons un cas de nerf laryngé inférieur droit non récurrent découvert à l'occasion d'une thyroïdectomie totale.

## Patient et observation

**Figure 1:**
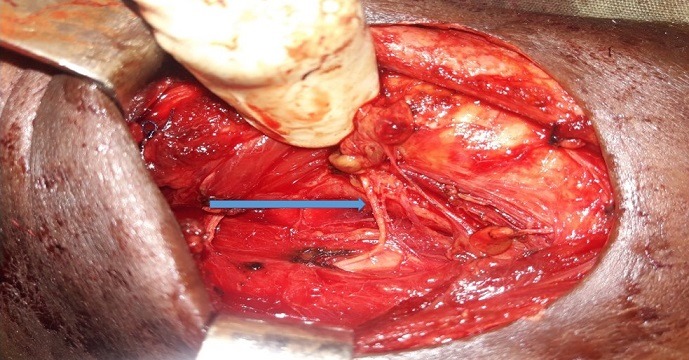
Le nerf laryngé inférieur non récurrent se détache du nerf vague et se dirige vers le larynx sans trajet récurrent

Il s'agissait d'une patiente de 32 ans, sans antécédents particuliers, suivie depuis 02 ans pour une maladie de Basedow. La chirurgie a été indiquée devant une absence de rémission de la maladie. Lors de la thyroïdectomie, le nerf laryngé inférieur droit n'avait pas été identifié ni derrière ni devant les branches de l'artère thyroïdienne inférieure droite ni à côté de la glande parathyroïde inférieure droite. On apercevait un filet blanc, semblant provenir en latéro-trachéal droit et perpendiculaire à la trachée, se dirigeant vers l'angle crico-trachéal droit (Figure 1). Ce filet se détachait du nerf vague droit et lorsque nous décidions de le suivre, nous nous apercevions qu'il rentrait dans l'angle crico-trachéal droit et correspondait au nerf laryngé inférieur droit. La poursuite de la thyroïdectomie s'était faite sans une autre surprise. A gauche, le nerf laryngé inférieur cheminait dans l'angle trachéo-oesophagien. Les suites opératoires ont été simples. La laryngoscopie indirecte effectuée en post opératoire montrait une bonne mobilité cordo-aryténoïdienne bilatérale.

## Discussion

Le nerf laryngé inférieur non récurrent est une variation anatomique rare et concerne exclusivement le côté droit à moins qu'il existe un *situs inversus* [2]. Toniato [3] rapporte une incidence de 0,51% parmi les cas de thyroïdectomie. Le nerf laryngé inférieur est classiquement identifié en utilisant les éléments de balisage que sont l'artère thyroïdienne inférieure et le bord inférieur du cartilage thyroïde. Mais, lorsque le repérage du nerf est difficile il faudrait procéder à sa palpation. En effet, à la palpation, le nerf ressemble à un cordon plaqué contre la trachée et situé entre l'artère carotide commune, l'artère thyroïdienne inférieure et la trachée [3,4]. Lorsque le nerf n'est toujours pas identifié malgré l'utilisation correcte de ses éléments de balisage, il faudrait alors avoir en tête la possibilité d'un nerf laryngé inférieur non récurrent. La dissection puis la mobilisation du nerf vague pourrait nous « montrer » le trajet du nerf récurrent laryngé [5]. Logiquement, une dissection rétrograde du nerf depuis son entrée dans l'angle crico-trachéal pourrait faciliter l'identification du nerf. D'autres auteurs ont proposé une identification des patients présentant un nerf laryngé inférieur non récurrent par l'échographie Doppler du tronc artériel brachio-céphalique. En effet, cette anomalie du côté droit est la conséquence d'une anomalie embryologique du développement des arcs branchiaux, comme l'attestent l'absence d'artère brachiocéphalique et la présence d'une artère subclavière aberrante (arteria lusoria) [6]. Par ailleurs, le monitoring du nerf laryngé constitue une méthode sûre, simple et efficace pour la surveillance peropératoire au cours d'une chirurgie de la thyroïde ou de la parathyroïde [7].

## Conclusion

Le nerf récurrent laryngé est un danger de la chirurgie thyroïdienne. Si le nerf n'a pas été découvert malgré l'utilisation de ses points de balisage, l'hypothèse d'un nerf laryngé inférieur non récurrent ne doit pas être écartée. Il convient alors de rechercher le nerf à son entrée dans le larynx et de le disséquer de façon rétrograde.

## Conflits des intérêts

Les auteurs ne déclarent aucun conflit d’intérêts.
